# Treatment of Primary Hypothyroidism by Slow-Release Liothyronine Monotherapy

**DOI:** 10.2174/0118715303321830241227112420

**Published:** 2025-01-21

**Authors:** Fereidoun Azizi, Atieh Amouzegar, Hengameh Abdi, Safdar Masoumi, Ladan Mehran

**Affiliations:** 1 Endocrine Research Center, Research Institute for Endocrine Sciences, Shahid Beheshti University of Medical Sciences, Tehran, Iran

**Keywords:** Hypothyroidism, clinical trial, levothyroxine, slow-release liothyronine, monotherapy, L-T4, SRT3 monotherapy

## Abstract

**Background:**

Combination therapy with levothyroxine (L-T4) and slow-release T3 (SRT3) in the treatment of hypothyroidism results in a normal triiodothyronine/thyroxine (T3/T4) ratio above that of L-T4 monotherapy. No clinical study has been reported on SRT3 monotherapy for hypothyroidism.

**Methods:**

This study was conducted in two parts. In the first part, 20 patients with primary hypothyroidism and serum thyrotropin (TSH) >30 mU/L were randomized into three groups receiving 1.6 μg/kg L-T4, equivalent doses of SRT3 or L-T3 of 0.55 μg/kg for 4 weeks. Their fasting serum-free T4 (fT4), T3, and TSH were measured weekly before taking medication for up to 4 weeks. In the second part, in 9 hypothyroid patients on L-T4 therapy and normal serum TSH, L-T4 therapy was discontinued, and a once-daily dose of SRT3 of 0.55 μg/kg was replaced. Serum fT4, T3, and TSH were measured weekly.

**Results:**

In part one, in patients treated with L-T3 and L-T4, serum TSH decreased to normal values after 4 weeks of intervention. In 7 patients on SRT3, serum T3 increased from 47 ± 12 at baseline to 110 ± 16 ng/dL, and serum TSH decreased from 60 ± 11 at baseline to 24 ± 10 and 26 ± 7 mU/L, respectively, at 14 and 21 days after intervention. At the end of 28 days, mean serum T3 was 110 ± 16, 168 ± 74, and 96 ± 18 ng/dL in SRT3, L-T3, and L-T4 groups, respectively (*p* < 0.001). In part two, serum fT4 decreased from 1.43 ± 0.7 to 0.41 ± 0.14 ng/dl, and serum T3 increased from 86 ± 21 to 113 ± 27 ng/dL by 21 days. Mean serum TSH remained normal until 14 days but increased to 15.1 ± 7.6 mU/L at 21 days. In the end, mean serum fT4, T3, and TSH were 0.35 ± 0.17 ng/dl, 77.4 ± 8.9 ng/dL, and 35 ± 11 mU/L, respectively.

**Conclusion:**

In patients with primary hypothyroidism, SRT3 monotherapy with an equivalent dose to L-T4 maintained normal serum T3 but could not sustain normal serum TSH concentration.

**Clinical Trial Registration Number:**

IRCT20100922004794N12.

## 1 INTRODUCTION

Hypothyroidism has been treated with replacement therapy, initially using a desiccated thyroid gland from 1891 and using synthetic levothyroxine (L-T4) since the 1950s [1-4].

L-T4 monotherapy is the current standard of care for hypothyroidism, as it maintains an adequate serum pool of T4 and provides triiodothyronine (T3) availability at cellular levels due to its deiodination in peripheral tissues [5-11]. It is also believed that a serum thyrotropin (TSH) within the normal range during L-T4 treatment indicates a state of euthyroidism at the hypothalamic-pituitary axis level and denotes adequate replacement therapy [12-14].

Recent data have reported that many hypothyroid patients on L-T4 monotherapy complain of hypothyroid symptoms despite a normal serum TSH concentration [15-17]. A landmark animal study showed that euthyroidism cannot be restored in plasma and all tissues of thyroidectomized rats on L-T4 monotherapy [18]. Other studies demonstrated that the T3/T4 ratio is decreased, psychological well-being and resting energy expenditure may be impaired, and body weight and serum lipid concentration may be higher in patients treated with L-T4 monotherapy than in control subjects [19-25]. In addition, it has been suggested that thyroid hormone levels may have stronger associations with clinical parameters than TSH levels [26].

Clinical trials using L-T4+L-T3 combination therapy showed increased serum fT3/fT4 ratio; however, most studies and all meta-analyses failed to show the superiority of this method over L-T4 monotherapy in resolution of hypothyroid symptoms and improvement of depression scale or quality of life outcomes [27-32]. The effectiveness and safety of L-T3 thrice daily have been observed, but it may not be practical in the long-term treatment of chronic diseases, such as hypothyroidism [33]. One study using a combination of slow-release T3 (SRT3) and L-T4 has reported a better T3/T4 ratio and a plateau in serum T3 up to 6 hours after ingestion of SRT3 [34]. We have also reported adequate normal serum T3 and T3/T4 ratios using a new formulation of SRT3 [35]. Although progress has been made in the formulation of SRT3 preparations, no clinical study has been reported on SRT3 monotherapy for hypothyroidism [36].

The present study aimed to evaluate the effectiveness of sustaining TSH normalization and the safety of the SRT3 product in treating hypothyroid patients.

## 2 MATERIALS AND METHODS

### 2.1 Study Design

This study was performed according to the ethical principles of the Helsinki Declaration of 1975, as revised in 2013, and all procedures on the study subjects were approved by the National Research Council of the Islamic Republic of Iran, the Human Research Review Committee of the Endocrine Research Center, Shahid Beheshti University, Tehran, Iran, (IR.SBMU.ENDOCRINE.REC.1402.031). The trial was registered in the Iranian Registry of Clinical Trials (www.irct.ir) with registration number ID: IRCT20100922004794N12. Trial participants signed informed consent forms at baseline, and their personal information will remain strictly confidential.

**Fig. (1):**
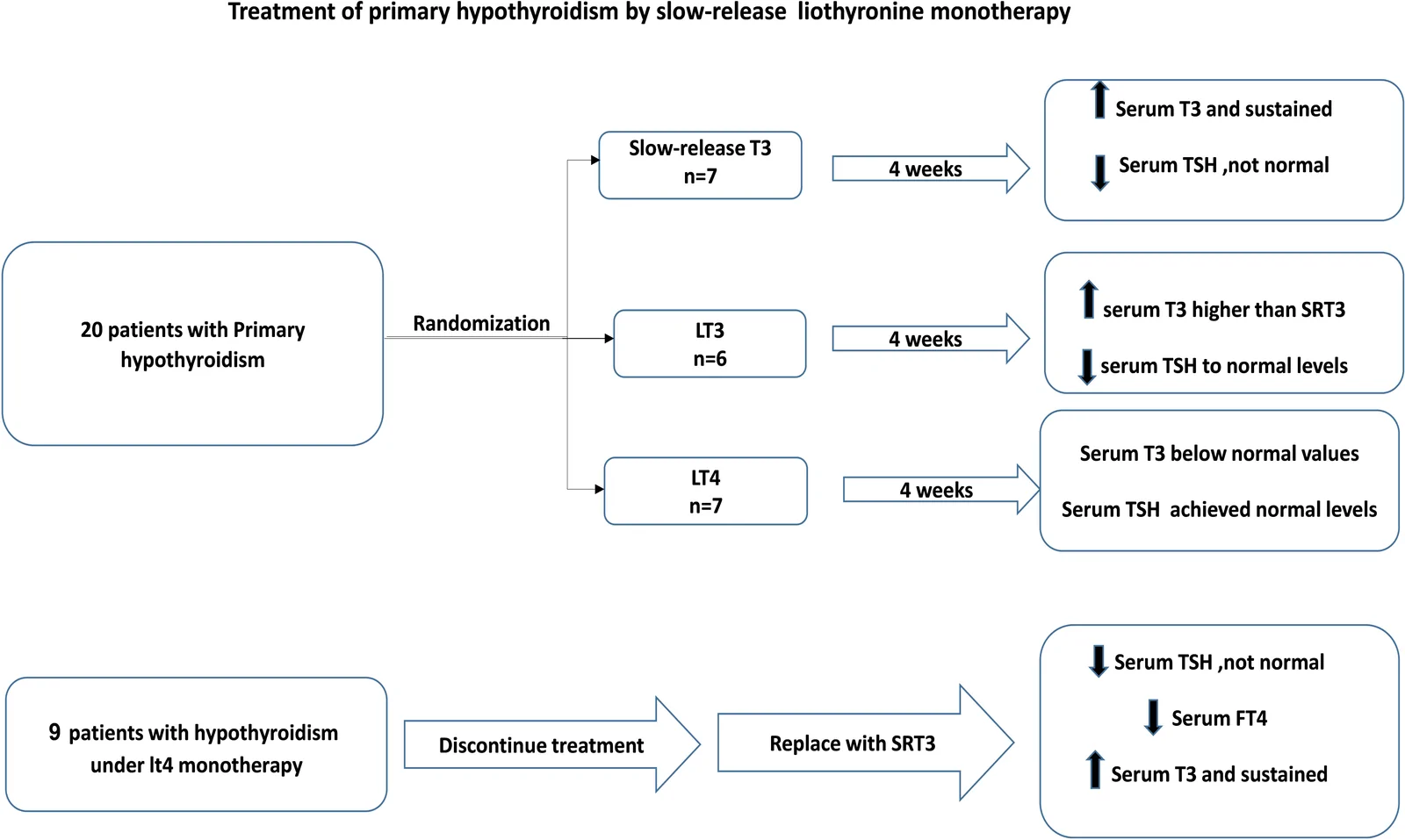
Flow chart of the allocating treatment and measurements in the study population. (**A**), 20 patients with primary hypothyroidism and serum TSH>30 mU/L were randomized into 3 groups receiving 1.6 μg/kg L-T4, equivalent doses of SRT3 or L-T3 of 0.55 μg/kg for 4 weeks, and their fasting serum fT4, T3, and TSH were measured weekly for up to 4 weeks. (**B**), in 9 hypothyroid patients on L-T4 therapy and normal serum TSH, L-T4 therapy was discontinued, and a once-daily dose of SRT3 0.55 μg/kg was replaced. Serum fT4, T3, and TSH were measured weekly.

The study is composed of two parts (Fig. **1**):

Part one: The effect of SRT3 monotherapy in patients with primary hypothyroidism

Twenty patients with primary hypothyroidism were included in this part of the study. Inclusion criteria were: no replacement therapy for at least two months before admission, serum T4 <0.6 ng/dL and TSH>30 mU/L, and body mass index (BMI) between 20-30 kg/m^2^. Exclusion criteria were pregnancy, lactation, cardiovascular, hepatic or renal disease, history or symptoms compatible with psychosis and major depression, and use of medications known to influence thyroid status or pharmacokinetics of thyroid hormones. After simple randomization, participants were assigned to a pre-breakfast regimen of SRT3, L-T3, or L-T4 for 4 weeks. The dose of L-T4 was 1.6 μg/kg, whereas the dose of SRT3 and L-T3 was 0.55 μg/kg. This dosage was chosen to attain a ratio of 0.34 for microgram/microgram LT3 to LT4 [35]. The medications were taken once daily, at least 30 minutes before breakfast. Patients returned to the endocrine clinic for weekly follow-up visits, and fasting blood sampling was done before taking the medication.

Part two: The effect of SRT3 monotherapy in the maintenance of normal serum TSH in L-T4 treated hypothyroid patients

Nine patients with primary hypothyroidism were allocated to this part of the study. Inclusion and exclusion criteria were: at least one-year duration of hypothyroidism with adequate L-T4 monotherapy, serum TSH concentration of 0.5-5.0 mU/L in at least two successive measurements before admission, and BMI of at least 20 and no higher than 30 kg/m^2^. Exclusion criteria were similar to the first part of the study. L-T4 therapy was discontinued in all nine patients, and SRT3 of 0.55 μg/kg, as the daily pre-breakfast regimen, was replaced. Patients returned to the endocrine clinic for weekly follow-up visits and blood sampling, as described in part one of this study.

### 2.2 Clinical Evaluation

At baseline and each weekly visit, symptoms and signs related to the study protocol were assessed. Adherence to the drug regimen was assessed by direct questioning and pill counting. Before the medication was taken, a fasting blood sample was obtained to measure serum fT4, T3, and TSH concentrations. The pills were ingested at least 30 minutes before breakfast. Patients were advised to continue their diet and avoid food and medications interfering with their absorption of thyroid hormones.

Safety was assessed on Treatment-Emergent Adverse Events (TEAE) observed by the researcher or reported by the patient spontaneously or after questioning. We also searched for all symptoms and signs of hyper- and hypothyroidism and cardiac arrhythmia in each visit.

### 2.3 Medications

L-T4 and L-T3 preparations of Iran Hormone Company have been validated locally, and L-T4 is the choice for treating hypothyroidism in Iran [37]. A sustained-release liothyronine sodium (SRT3) formulation was developed by Noor Research and Education Institute (Tavan), and its pharmacodynamics and pharmacokinetic properties were previously reported [35].

### 2.4 Laboratory Measurements

All laboratory measurements were determined on -20°C stored serum samples. Thyroid function tests, including TSH, T3, and fT4, were measured by electrochemiluminescence immunoassay method using Roche Diagnostics kits on the Cobas e-411 automated analyzer (Roche Diagnostics, GmbH, Mannheim, Germany). The sensitivity of TSH, T3, and fT4 assays was 0.005 mU/L, 0.195 ng/mL, and 0.5 pmol/L, respectively. Lyophilized quality control materials (Lyphochek Immunoassay Plus Control, Bio-Rad Laboratories) in three different concentrations were used to monitor the accuracy of measurements. The intra- and inter-assay coefficient of variations (CVs) were 2.8% and 3.1%, respectively.

Thyroid hormone reference ranges for the Tehranian population are fT4 0.9-1.55 ng/dL, T3 75-200 ng/dL, and TSH 0.32-5.06 mU/L [38].

### 2.5 Statistical Analysis

Quantitative variables were reported using mean and standard deviation. To assess changes in fT4, T3, and TSH levels from baseline to days 7, 14, 21, and 28, a generalized estimating equation (GEE) model was used. Mean differences and 95% confidence intervals (CIs) were calculated to determine changes from the baseline. A confidence interval not including zero or a *P*-value less than 0.05 was considered statistically significant. All analyses were performed using Stata software (StataCorp. 2015. Stata Statistical Software: Release 14. College Station, TX: StataCorp LP).

## 3 RESULTS

### 3.1 Part One

All twenty patients with hypothyroidism adhered to the allocated therapy until the end of the study. Table **1** demonstrates the baseline characteristics of the three study groups. There was no difference in age, sex, BMI, duration of hypothyroidism, and serum concentrations of fT4, T3, and TSH among the study groups at baseline.

**Fig. (2):**
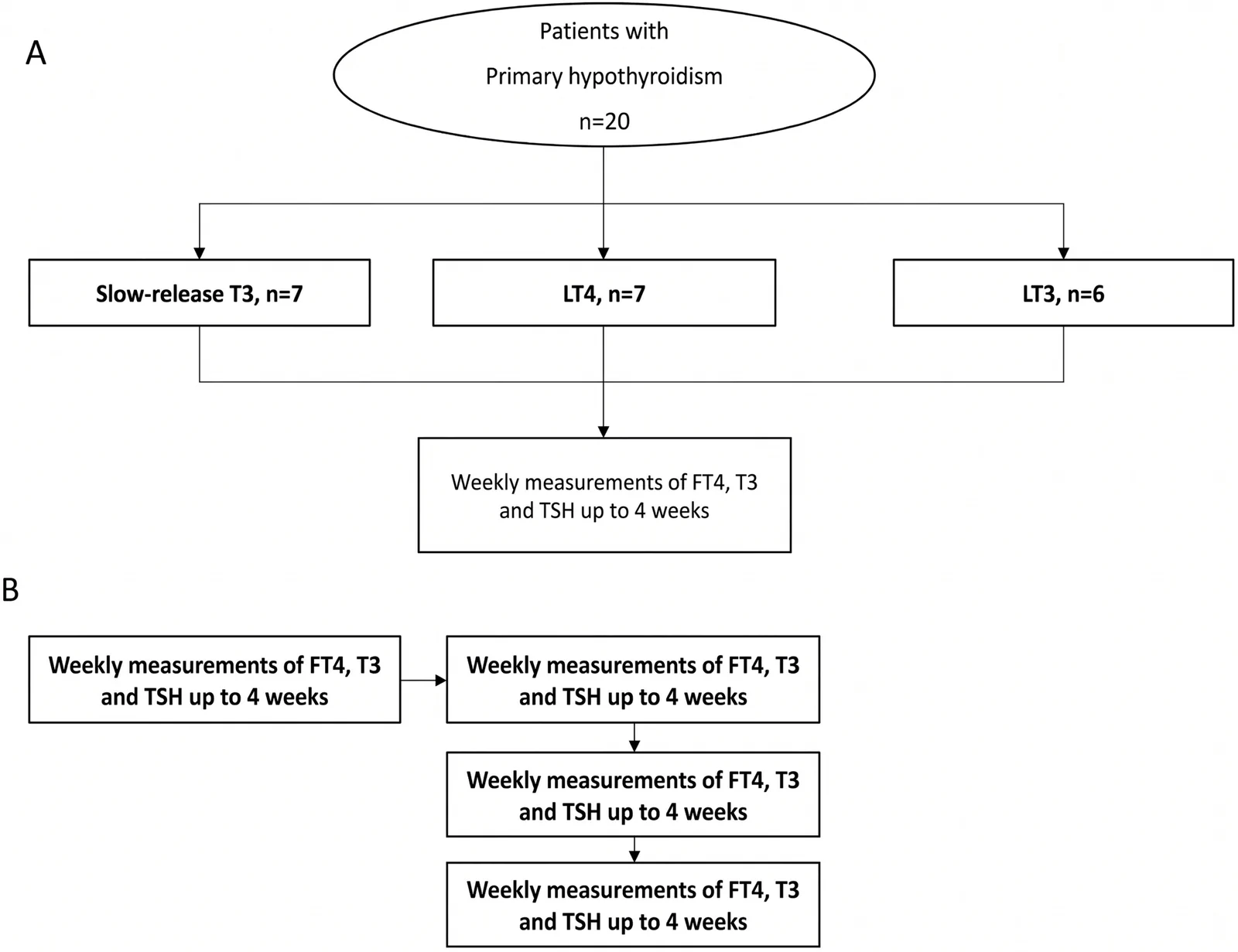
Changes in serum concentration of fT4, T3, and TSH and T3/fT4 ratio after treatment with SRT3, L-T3, and L-T4 medications in patients with primary hypothyroidism.

As expected, serum fT4 and T3 increased, and serum TSH levels decreased in hypothyroid patients receiving L-T4 therapy. In patients treated with L-T3, serum fT4 concentration remained low. In contrast, serum T3 increased, and serum TSH decreased to normal values (Fig. **2**). At the end of the study, the mean serum concentration of T3 in the L-T3 treated group was significantly higher than in the other two groups (168 ± 74 *vs*. 96 ± 18 ng/dL in L-T4 group and 110 ± 16 ng/dL in the SRT3 group) (*p* < 0.001).

In 7 patients treated with a once-daily dose of SRT3, compared to baseline values, there was a significant increase in serum T3 (87.8 ± 15.9 *vs* 47.4 ± 11.7 ng/dL, *p* < 0.001) after 7 days of therapy. Mean serum fT4 remained low, and serum TSH decreased significantly in all seven patients (35.0 ± 10.8 *vs* 60.7 ± 10.8 mU/L, *p* < 0.001). The increase in serum T3 and a decrease in serum TSH continued during the second week of the study. By 14 days of SRT3 usage, serum T3 increased to 107.5 ± 17.7 ng/dL, and serum TSH decreased to 23.7 ± 10.1 mU/L (Fig. **2**). Continuation of SRT3 monotherapy up to 4 weeks resulted in sustained serum levels of T3 and TSH with concentrations significantly different from the baseline values (*p* < 0.001), but not significantly different from those of 14 days of therapy (Table **2**).

### 3.2 Part two

Baseline mean serum concentrations of fT4, T3, and TSH in 9 hypothyroid patients on 112 ± 21 μg of L-T4 daily were 1.43 ± 0.27 ng/dL, 85.9 ± 20.8 ng/dL and 2.21 ± 1.84 mU/L, respectively (Table **3**). Levothyroxine was discontinued, and 7 days after SRT3 treatment, mean serum T4 decreased to 0.85 ± 0.24 ng/dL, mean serum T3 increased to 130.1 ± 12.2 ng/dL, and mean serum TSH remained in normal values in all nine patients with a mean of 2.7 ± 18 mU/L. Differences in serum fT4 and T3 concentrations between baseline and 7 days after intervention were significant (*p* < 0.001).

At 14 days after the intervention, there was a further decrease in mean serum fT4 values to 0.63 ± 0.11 ng/dL (*p* < 0.001, compared to baseline). Mean serum T3 was significantly higher as compared to baseline (120.2 ± 7.9 *vs.* 85.9 ± 20.8 ng/dL, *p* < 0.001) but not significantly different from serum T3 at 7 days after the intervention. Mean serum TSH was non-significantly higher than baseline values; however, TSH was higher than normal in six of nine patients, which would indicate that these patients were replaced, Serum fT4 decreased further at 21 and 28 days after discontinuation of L-T4 therapy. At day 21 of intervention, mean serum T3 decreased to 112.5 ± 26.7 ng/dL, not statistically different from values at 7 and 14 days of study, and mean serum TSH increased to 15.15 ± 7.63 mU/L, significantly higher than baseline values (*p* < 0.001). After 28 days of intervention, serum T3 decreased further to 77.4 ± 8.9 ng/dL, not significantly different from baseline, while the levels of 8 of 9 patients were still within normal limits, and mean serum TSH increased to 35.44 ± 11.50 mU/L (*p* < 0.001) (Table **3**).

### 3.3 Safety

No severe side effects, such as cardiovascular complications, including arrhythmia, were observed in patients of the three groups. During part one of the study, weakness and fatigue occurred in 5, 2, and 4 patients, and pulse was detected in 0, 3, and 1 patients in SRT3, L-T3, and L-T4 groups, respectively. Only two patients complained of fatigue and weakness after 4 weeks of treatment with SRT3 in part two of the study, even though all subjects had elevated TSH levels.

## 4 DISCUSSION

To the best of our knowledge, this is the first report of a clinical trial of SRT3 monotherapy in patients with primary hypothyroidism. Clinical trials using L-T3 and L-T4 monotherapy have frequently been performed, and their pharmacodynamic and pharmacokinetic properties have been reported [1, 6, 39-45]. The present study showed that SRT3 monotherapy with an equivalent dose to L-T4 maintains normal serum T3 but cannot sustain normal serum TSH concentration.

In the brain, the exchange of T3 with serum is slow, and most of the T3 is produced *in situ*; therefore, fluctuations in the rate of T3 degradation have a more significant influence on tissue concentrations of T3, compared to tissues, such as the liver and kidney, in which cellular T3 is in more rapid equilibration with serum [46-52]. In the pituitary gland, additional T3 is provided by intracellular T4 to T3 conversion, and thyroid receptors occupied by locally generated T3 are much higher than T3 provided from blood [53, 54]. Furthermore, normal serum levels of T3 and T4 are required to suppress TSH and RNA production in the hypothalamus's periventricular nucleus and normalize serum TSH [55-63]. Although administration of a single dose of T3 has minimal effect on reducing serum TSH concentration [64-66], daily administration of L-T3 normalizes serum TSH in hypothyroid patients [33]. The precise time sequence of inhibition in TRH responsiveness depends on how much the level of T3 in the blood increases [67-70]. Hence, during monotherapy with L-T3, a significantly higher T3 serum concentration is required to maintain serum TSH within normal levels [71, 72].

The results of the present study regarding L-T3 and L-T4 monotherapy are in agreement with the previous reports [6, 27, 29, 73]. We used the 0.34 μg ratio of L-T3/L-T4 dose according to the earlier estimates obtained by compartmental analysis [74] and the substitution studies of L-T3 for L-T4 at equivalent doses [33]. L-T4 monotherapy resulted in lower serum T3 and T3/ fT4 ratio, and L-T3 monotherapy caused higher serum T3 and T3/fT4 ratio. This is not surprising since the pituitary relies in part on the intracellular conversion of T4 to maintain adequate levels of T3 [37, 38].

Our findings of SRT3 monotherapy in patients with hypothyroidism clearly demonstrated that the 0.34 μg ratio of SRT3/L-T4 dose was not able to maintain serum TSH levels within the normal range despite achieving normal serum T3 levels; however, the mean serum T3 achieved by this dose of SRT3 was below the mean serum T3 in normal subjects. The mean serum T3 was 130.1 ± 12.2, 120.2 ± 7.9, and 112.5 ± 26.7 ng/dl at 7, 14, and 21 days after SRT3 monotherapy but declined further to 77.4 ± 8.9 ng/dl at 28 days of therapy. This finding indicated that a good part of serum T3 in the first 3 weeks of the study came from the deiodination of previous L-T4 treatment. When L-T4 therapy was discontinued, this part decreased, and the part of T3 increased due to SRT3 therapy dropped by 4 weeks. In addition, L-T3 monotherapy in equal dosage resulted in much higher serum T3 levels than SRT3 monotherapy and could maintain normal TSH concentrations. Therefore, higher doses of SRT3 are needed to achieve the euthyroid state. This fact was also evident in the second part of the study. In hypothyroid patients who had achieved euthyroidism with L-T4 monotherapy, discontinuation of L-T4 and substitution of equivalent SRT3 dosage caused 30-40% increase in serum T3 concentration in the first 2 weeks of study; however, with gradual decrease in serum fT4 levels and fall in T4 to T3 conversion, serum concentration of T3 decreased in the following weeks and lack of generation of T3 from T4 in the pituitary caused elevation of serum TSH concentration. Therefore, higher doses of SRT3 are needed to achieve the euthyroid state and suppression of serum TSH, which may introduce the possibility of risks associated with higher hormone levels.

The 2012 ETA Guidelines on the use of L-T4+L-T3 in the treatment of hypothyroidism suggested a dose ratio between 13:1-20:1 by weight [16]. However, in the study of Hennemann *et al.*, using a combination ratio of 1:20 of SRT3+L-T4, the mean T3/T4 ratio did not reach normal values [34]. The present findings emphasize that a higher ratio of STR3 is needed in combination with treatment to obtain a normal T3/T4 ratio, as suggested in our previous study [22].

The strength of this study is that we aimed, designed, and executed the study to address a knowledge gap in terms of the comparative effectiveness of SRT3 *versus* L-T3 and L-T4 therapies for treating primary hypothyroidism. It is important to emphasize that all thyroid hormone measurements were done over fasting and before drug ingestion, making the results more consistent. The findings may be helpful in further investigations of SRT3 monotherapy.

This study has a few limitations. First, the sample size is very small in both parts of the study, which restricts the generalizability of the findings to a broader population. Second, the duration of the trial is short. The first part of the study lasted only 4 weeks, which is relatively short for assessing the long-term efficacy and safety of SRT3 monotherapy. This duration of treatment may not capture all potential outcomes and adverse effects. However, the inability of SRT3 to further decrease serum TSH from 14 to 28 days of study suggests that even with continuous treatment of a similar dosage of SRT3, serum T3 may not be achieved. Third, the present trial aimed to assess the effect of SRT3 on serum hormone levels; future studies should evaluate the broader physiological and psychological impacts of this preparation of peripheral tissues. Fourth, although short-term safety was evaluated in this trial, further studies are needed to explore the long-term adverse events, such as cardiovascular diseases associated with thyroid hormone therapies [75].

## CONCLUSION

The results of this study demonstrate the ability of SRT3 preparation to maintain normal serum T3 levels. However, the equivalent doses of this product are not able to maintain serum TSH concentration within normal ranges in patients with primary hypothyroidism not taking levothyroxine. Further studies with large number of participants, varying doses of SRT3, and longer duration of therapy will be necessary to determine various aspects of treatment with SRT3 preparations.

## Figures and Tables

**Table 1:** Baseline characteristics of the hypothyroid participants randomly allocated to three groups under treatment with SRT3, LT3, and L-T4.

-	**Treatment Allocation**		
	**SRT3**	**L-T3**	**L-T4**
Age (year)	50 ± 7	49 ± 8	49 ± 10
Female/Male	6/1	6/1	5/1
Body Mass Index (kg/m^2^)	25.1 ± 3.8	26.0 ± 4.1	25.5 ± 3.7
Duration of hypothyroidism (Y)	4.6 ± 8.2	5.3 ± 9.21	4.9 ± 7.6
fT4 (ng/dL)	0.56 ± 0.23	0.40 ± 0.14	0.49 ± 0.13
T3 (ng/dL)	51 ± 11	54 ± 14	5.6 ± 14
TSH (mU/L)	65 ± 15	61 ± 11	59 ± 13

**Abbreviations:** SRT3: Slow release triiodothyronine; L-T3: Liothyronine; L-T4: Levothyroxine. **Note:** Values are expressed as mean ± SD.

**Table 2:** Serum concentrations of fT4, T3, and TSH in 7 patients with primary hypothyroidism at baseline and 7, 14, 21, and 28 days of treatment with SRT3.

-	**No.**	**Baseline**	**7 Days**	**14 Days**	**21 Days**	**28 Days**
fT4 (ng/dL)	1	0.4	0.4	0.4	0.4	0.4
	2	0.3	0.3	0.3	0.3	0.3
	3	0.7	0.6	0.4	0.4	0.4
	4	0.8	0.7	0.6	0.6	0.5
	5	0.6	0.5	0.4	0.3	0.3
	6	0.7	0.6	0.5	0.4	0.4
	7	0.4	0.4	0.4	0.4	0.3
	Mean ± SD	0.56 ± 0.19	0.50 ± 0.14	0.43 ± 0.10	0.40 ± 0.10	0.37 ± 0.08
	Mean Diff (95% CI)	Reference	-0.06 (-0.12 to 0.01)	-0.13 (-0.20 to -0.06)	-0.16 (-0.22 to -0.09)	-0.19 (-0.25 to -0.12)
	*P* value	-	0.09	**<0.001**	**<0.001**	**<0.001**
T3 (ng/dL)	1	46	105	125	145	138
	2	39	62	75	100	82
	3	35	78	98	105	110
	4	70	92	100	110	115
	5	54	100	120	115	108
	6	41	77	120	95	105
	7	47	101	115	108	112
	Mean ± SD	47.4 ± 11.7	87.8 ± 15.9	107.5 ± 17.7	111.1 ± 16.3	110.0 ± 16.4
	Mean Diff (95% CI)	Reference	40.43 (30.41 to 50.45)	60.14 (50.12 to 70.17)	63.71 (53.69 to 73.74)	62.57 (52.55 to 72.59)
	*P* value	-	**<0.001**	**<0.001**	**<0.001**	**<0.001**
TSH (mU/L)	1	60	20	9.3	18	12
	2	53	41	35	30	30
	3	75	40	32	22	27
	4	42	35	30	26	22
	5	62	28	12	21	19
	6	70	53	20	33	34
	7	63	28	28	25	26
	Mean ± SD	60.7 ± 10.8	35.0 ± 10.8	23.7 ± 10.1	25.0 ± 5.23	24.3 ± 7.32
	Mean Diff (95% CI)	Reference	-25.71 (-32.78 to -18.65)	-36.96 (-44.02 to -29.89)	-35.71 (-42.78 to -28.65)	-36.43 (-43.49 to -29.37)
	*P* value	-	**<0.001**	**<0.001**	**<0.001**	**<0.001**

**Abbreviations:** TSH, thyroid-stimulating hormone; T3, triiodothyronine; FT4, free thyroxine; **Note:*** P*-value was calculated using Generalized Estimating Equations.

**Table 3:** Serum concentrations of fT4/T3 and TSH concentrations in 9 patients with hypothyroidism on levothyroxine at baseline and their changes 7, 14, 21, and 28 days after withdrawal of L-T4 and treatment with SRT3.

-	-	**Baseline** **(On L-T4)**	**Treatment with Slow-release T3**			
	**No.**		**7 Days**	**14 Days**	**21 Days**	**28 Days**
fT4 (ng/dL)	1	1.8	1	0.6	0.3	0.2
	2	1.4	0.9	0.6	0.7	0.5
	3	1.4	1.1	0.8	0.5	0.5
	4	1.6	0.9	0.5	0.2	0.2
	5	1.4	1	0.7	0.4	0.2
	6	0.9	0.4	0.8	0.5	0.7
	7	1.2	0.5	0.6	0.4	0.3
	8	1.7	0.9	0.5	0.4	0.3
	9	1.5	1	0.6	0.3	0.3
	Mean ± SD	1.43 ± 0.27	0.85 ± 0.24	0.63 ± 0.11	0.41 ± 0.14	0.35 ± 0.17
	Mean Diff (95% CI)	Reference	-0.58 (-0.76 to -0.41)	-0.81 (-0.98 to -0.63)	-1.03 (-1.21 to -0.85)	-1.09 (-1.26 to -0.91)
	*P* value	-	**<0.001**	**<0.001**	**<0.001**	**<0.001**
T3 (ng/dL)	1	93.0	147.0	122.0	67.0	62.0
	2	100.0	125.0	132.0	147.0	80.0
	3	100.0	130.0	128.0	140.0	70.0
	4	90.0	110.0	110.0	90.0	90.0
	5	81.0	122.0	115.0	125.0	85.0
	6	70.0	130.0	120.0	113.0	80.0
	7	115.0	138.0	125.0	88.0	85.0
	8	81.0	122.0	108.0	110.0	75.0
	9	43.0	147.0	122.0	133.0	70.0
	Mean ± SD	85.9 ± 20.8	130.1 ± 12.2	120.2 ± 7.9	112.5 ± 26.7	77.4 ± 8.9
	Mean Diff (95% CI)	Reference	44.22 (29.13 to 59.32)	34.33 (19.24 to 49.43)	26.67 (11.57 to 41.76)	-8.44 (-23.54 to 6.65)
	*P* value	-	**<0.001**	**<0.001**	**0.001**	0.27
TSH [mU/L]	1	0.8	0.5	5.7	29.1	47.0
	2	4.4	3.9	6	14.4	28.0
	3	2.4	4.2	7.2	14.0	31.0
	4	0.5	3.3	0.5	4.8	53.0
	5	4.1	3.5	6.2	14.1	50.0
	6	0.9	2.8	1.2	15.0	20.0
	7	0.8	0.6	5.2	24.0	31.0
	8	5.1	5.0	7.0	15.0	30.0
	9	0.9	0.8	4.0	6.0	29.0
	Mean ± SD	2.21 ± 1.84	2.73 ± 1.68	4.77 ± 2.42	15.15 ± 7.63	35.44 ± 11.50
	Mean Diff (95% CI)	Reference	0.52 (-5.01 to 6.06)	2.57 (-2.97 to 8.10)	12.94 (7.41 to 18.48)	33.23 (27.70 to 38.77)
	*P* value	-	0.85	0.36	**<0.001**	**<0.001**

**Abbreviations:** TSH, thyroid-stimulating hormone; T3, triiodothyronine; FT4, free thyroxine. **Note:** *P* value was calculated using Generalized Estimating Equations.

## Data Availability

Some or all datasets generated during and/or analyzed during the current study are not publicly available but are available from the corresponding author on a reasonable request.

## LIST OF ABBREVIATIONS

L-T4: Levothyroxine
T3/T4: Triiodothyronine/thyroxine
fT4: Serum-free T4
GEE: Generalized Estimating Equation
CIs: Confidence Intervals

## STANDARDS OF REPORTING

CONSORT guidelines were followed.
